# Applying real-world data from expanded-access (“compassionate use”) patients to drug development

**DOI:** 10.1017/cts.2023.606

**Published:** 2023-08-07

**Authors:** June S. Wasser, David J. Greenblatt

**Affiliations:** From the Clinical and Translational Science Institute, Tufts Medical Center and Tufts University School of Medicine, Boston, MA, USA

**Keywords:** Real-world data, compassionate use, expanded access, 21st Century Cures Act, drug development

## Abstract

Our drug development process has produced many life-saving medications, but patients experiencing rare diseases and similar conditions often are left with limited options for treatment. For an approved treatment to be developed, research on a new candidate or existing drug must validate safety and efficacy based on contemporary research expectations. Randomized clinical trials are conducted for this purpose, but they are also costly, laborious, and time-consuming. For this reason, The 21^st^ Century Cures Act mandates that the US Food and Drug Administration look for alternative methods for approving drugs, in particular exploring the uses of real-world data and evidence. Expanded access (“compassionate use”) is a pathway for the clinical treatment of patients using drugs that are not yet approved for prescribing in the United States. Using real-world evidence generated from expanded-access patients presents an opportunity to provide critical data on patient outcomes that can serve regulatory approval in conjunction with other observational datasets or clinical trials, and in limited circumstances may be the best data available for regulatory review. In doing so, we may also support and encourage patient-centered care and a personalized medicine approach to drug development.

## Introduction

The treatment pathway for patients experiencing a dire and unmet medical need is universally known as compassionate use, or as defined by the US Food and Drug Administration (FDA), expanded access (EA). Other terms used to describe the EA process include: preapproval access, early access, managed access, and named-patient and single-patient IND. The first EA FDA regulation for an emergency single-patient IND was introduced in the 1970s, and expanded-access programs for more than one patient were created to address the acquired immunodeficiency syndrome epidemic in a 1987 multi-patient FDA guidance [1]. A broader guidance was released in 2009 defining single patient, intermediate-size group, and larger treatment protocols [2]. The commonality of all patients, regardless of their single or group status, is that these patients have exhausted all approved treatment options and are not eligible for any ongoing clinical trials, and no comparable or alternate treatment exists.

EA was established as treatment for critically ill patients and not a research pathway, with recognition that data from any type of patient care is valuable to the care of future patients, thereby emphasizing the indistinct lines between treatment and research. In a more ideal health care system that incorporated greater adaptability and flexibility, treatment and research would be more readily recognized as part of an iterative process, such that data we can capture that enables learning about treatment effects and outcomes is not only important to the health system, but in this case, important to the drug development process.

Randomized clinical trials are the gold standard by which the FDA evaluates whether a new drug is effective and safe to enter the US market, but increasingly other forms of data are used by FDA and other regulatory agencies for post-market safety evaluation and for new drug submissions. These data types include electronic health records (EHR), medical claims data, and patient-reported outcomes. Like EA data, EHR and claims data were not originally collected for research purposes but over time they have evolved into a dual role. While EA data lack the rigor and standardization of clinical trial data, it offers for consideration a broader and more diverse pool of patients, more closely resembling a real-world setting. Such data can potentially supplement clinical trial data in the regulatory review process. EA data may also inform decisions regarding off-label use, cost reimbursement, and additional treatment indications.

Understanding how real-world data might be generated, collected, and used from treating patients with EA drugs can lead to a more scientific approach and development of best practices. For contextual purposes, real-world data are defined as observational data generated through clinical treatment. If we were to consider these circumstances through a research lens, it would most resemble an “N-of-1” study [3], with a single patient as the study population (as in a single-patient IND). Or it could consist of a group of patients treated under an EA program protocol with a traditional IND. Though EA is considered treatment, it is nonetheless important to recognize that this FDA pathway includes a patient-informed consent process and Institutional Review Board approval because the drug is investigational.

### Comment

While many EA drugs have historically been provided to oncology patients [4,5], the potential to advance drug development for rare disease patients is of special interest. There are over 7,000 rare diseases affecting 10% of the population, with very few existing treatment options [6]. Children make up half of these patients whose conditions are often life-threatening or life-limiting. Pharmaceutical sponsors are challenged by many of these diseases because of the limited number of patients available for study. Consequently, small patient populations make every dataset significant. This apparent disadvantage placed on both patients and drug developers might be addressed by using the EA process for treatment, data collection, and regulatory submission if aligned with FDA review. This could mean manufacturers engaging with FDA in early-stage drug development and discussing a streamlined approach to establishing EA data points [7].

One of the barriers to EA data collection is the additional cost and operational burden to sponsors. A specific example would be the resources needed to develop and implement standardized data collection systems to streamline regulatory review, and to enhance cross-national data sharing involving regulatory agencies from different regions of the world. For emerging or small manufacturers, the cost might be prohibitive. For large pharmaceutical and biotechnology manufacturers, systems that support their worldwide network and address the needs of multiple regulatory systems will facilitate the global objective of delivering medical care to the population of affected patients. The International Council for Harmonization, in existence since 1990, is a cross-national professional organization devoted to harmonizing the drug development process across nations [https://www.ich.org/] and has systems and guidelines in place that could facilitate the broadening of EA worldwide. Providing EA under expanded access protocols rather than single-patient INDs can be operationally more efficient and serve a greater number of patients. However, there is an important place for single-patient INDs. With appropriate harmonization of protocol structure, data collection procedures, and analysis strategies, the data from single patients can in principle still be aggregated to allow more generalizable inferences.

Increasingly, EA data is included in FDA new drug submissions. Examples of FDA drug approvals incorporating EA data include glucarpidase in 2012 [8,9]. It is a chemotherapy reversal agent to treat toxic plasma methotrexate concentrations in patients with impaired renal function. Clinical evidence included 22 patients in two efficacy studies and an EA protocol. Between 2017 and 2018, three other drug approvals included EA protocols: vestronidase to treat mucopolysaccharidosis VII, a rare genetic enzyme deficiency [10,11]; lutetium 177 dotatate injection, a radiolabeled drug for rare gastroenteropancreatic neuroendocrine tumors [12–14]; and cannabidiol, an adjunctive treatment for seizures associated with two rare conditions [15].

EA evidence alone was used in an additional four examples: combined sodium phenylacetate and sodium benzoate [16] to treat acute hyperammonaemia in patients with a rare urea cycle disorder; uridine triacetate [17] following overdoses with 5-fluorouracil or capecitabine; cholic acid [18,19] to treat bile acid disorders; and nitisinone [20–22] to treat hereditary tyrosinaemia type 1.

More recently, in April 2022, alpelisib received accelerated approval from the FDA for treating severe manifestations of PIK3CA-related overgrowth spectrum [23,24]. Patients received alpelisib through an EA program, and additional real-world data was collected through a retrospective medical chart review [25]. The use of this integrated data approach offers an example of a new and interesting model for clinical treatment, research, and regulatory approval – serving patients, drug developers, and the FDA.

## Conclusion

Both drug developers and regulatory authorities are searching for ways to better serve patients, especially those patient populations that may not have access to other treatment options. The traditional approach is for randomized clinical trials to serve a restricted group of patients who meet specific and relatively inflexible inclusion/exclusion criteria. This approach has demonstrated success and will continue to be the basis of most research studies, but opportunities exist to implement novel approaches to helping patients that do not meet clinical trial criteria, or may not be near an investigational trial site, or even worse are experiencing a medical crisis for which there is no ongoing trial because the disease is so rare that few patients are afflicted. In these instances, EA treatment may form the basis for a singularly existing potential treatment, and simultaneously present an important learning opportunity for drug development.

The creation of a new regulatory framework will encourage drug developers to identify additional opportunities for patient care, to reach critical groups of patients, and to also ensure that patients are served equitably and safely. The FDA guidance process is a means for the agency to share its current thinking on a given topic. Drug manufacturers depend on guidance documents as methods to advance their research and business practices, and will look to what specific types of evidence and standards the FDA will use to evaluate data generated from EA experience. In the case of rare diseases, the benefit/risk profile can be very different for a life-saving drug compared to a drug treating chronic illness. While the FDA has signaled an openness to accepting EA patient treatment data for regulatory purposes, creating a formal framework for this type of real-world evidence will help to stimulate similar regulatory submissions, and ensure that evidence is of the highest quality. It will also support clinicians and drug development in general by assessing the impact of personalized care.

Using real-world evidence derived from clinical treatment has the potential to create a new way of incorporating data that could inform manufacturers and regulatory authorities on important approaches, potentially accelerate drug development, and at the very least, make it more patient-centered.

## Related Regulatory Announcements

https://www.prnewswire.com/news-releases/fda-approves-voraxaze-to-treat-patients-with-toxic-methotrexate-levels-137519843.html.

https://www.fda.gov/news-events/press-announcements/fda-approves-treatment-rare-genetic-enzyme-disorder.

https://www.fda.gov/news-events/press-announcements/fda-approves-new-treatment-certain-digestive-tract-cancers.

https://www.fda.gov/news-events/press-announcements/fda-approves-first-drug-comprised-active-ingredient-derived-marijuana-treat-rare-severe-forms.

https://www.fda.gov/drugs/resources-information-approved-drugs/fda-approves-alpelisib-pik3ca-related-overgrowth-spectrum.

https://www.fdanews.com/articles/68953-medicis-announces-fda-approval-of-ammonul-for-urea-cycle-disorder.

https://cms.centerwatch.com/directories/1067-fda-approved-drugs/listing/4415-vistogard-uridine-triacetate.

https://cms.centerwatch.com/directories/1067-fda-approved-drugs/listing/3305-cholbam-cholic-acid.
